# Yeast Extract Stimulates Ginsenoside Production in Hairy Root Cultures of American Ginseng Cultivated in Shake Flasks and Nutrient Sprinkle Bioreactors

**DOI:** 10.3390/molecules22060880

**Published:** 2017-05-26

**Authors:** Ewa Kochan, Piotr Szymczyk, Łukasz Kuźma, Anna Lipert, Grażyna Szymańska

**Affiliations:** 1 Pharmaceutical Biotechnology Department, Medical University of Lodz, Muszyńskiego 1, Lodz 90-151, Poland; piotr.szymczyk@umed.lodz.pl (P.S.); grazyna.szymanska@umed.lodz.pl (G.S.); 2 Department of Biology and Pharmaceutical Botany, Medical University of Lodz, Muszyńskiego l, Lodz 90-151, Poland; lukasz.kuzma@umed.lodz.pl; 3 Department of Sports Medicine, Medical University of Lodz, Pomorska 251, Lodz 92-213, Poland; anna.lipert@umed.lodz.pl

**Keywords:** hairy root cultures, nutrient sprinkle bioreactor, yeast extract, elicitation

## Abstract

One of the most effective strategies to enhance metabolite biosynthesis and accumulation in biotechnological systems is the use of elicitation processes. This study assesses the influence of different concentrations of yeast extract (YE) on ginsenoside biosynthesis in *Panax quinquefolium* (American ginseng) hairy roots cultivated in shake flasks and in a nutrient sprinkle bioreactor after 3 and 7 days of elicitation. The saponin content was determined using HPLC. The maximum yield (20 mg g^−1^ d.w.) of the sum of six examined ginsenosides (Rb1, Rb2, Rc, Rd, Re and Rg1) in hairy roots cultivated in shake flasks was achieved after application of YE at 50 mg L^−1^ concentration and 3 day exposure time. The ginsenoside level was 1.57 times higher than that attained in control medium. The same conditions of elicitation (3 day time of exposure and 50 mg L^−1^ of YE) also favourably influenced the biosynthesis of studied saponins in bioreactor cultures. The total ginsenoside content was 32.25 mg g^−1^ d.w. and was higher than that achieved in control medium and in shake flasks cultures. Obtained results indicated that yeast extract can be used to increase ginsenoside production in hairy root cultures of *P. quinquefolium*.

## 1. Introduction

Elicitation is one of the most effective strategies used for enhancing the production of secondary compounds in in vitro cultures. This process consists of adding elicitors to the culture medium. Originally the term “elicitor” was used for molecules capable of the inducing phytoalexin production, but now it commonly refers to chemicals from various sources that can trigger defensive reactions in plants, leading to the accumulation of secondary metabolites. Elicitors are classified based on their “nature” as abiotic and biotic [1].

Abiotic elicitors (AE) have no biological origin and they include physical (UV radiation, usage of freeze-thaw cycles, osmotic stress, salinity, drought) and chemical (mineral salts, heavy metals and chemical compounds capable of DNA and cell membrane damage: detergents, fungicides and herbicides) factors [2]. Biotic elicitors (BE) are substances of biological origin. They may be derived from a pathogen (exogenous elicitors) or from the plant (endogenous elicitors). Exogenous BE are released by microorganisms and other pathogens or formed by the action of plant enzymes on microbial cell walls (e.g., fungal and bacterial lysates, yeast extracts, chitin, glucans). Endogenous biotic elicitors are polysaccharides originating from pathogen degradation of the plant cell wall, intracellular proteins and small molecules synthesized by the plant cell in response to different types of stress or pathogen attack. The efficiency of elicitation process depends primarily on the interaction between the plant cell and the elicitor. Furthermore, the parameters such as the type and concentration of the elicitor, the elicitation time exposure, the cell lines, the addition of growth regulators, the media and culture conditions have an influence on the elicitor activity [1].

One of the biotic elicitors that can result in the improvement of secondary metabolites content is yeast extract (YE) [3,4,5,6]. Like other elicitors, YE concentration in the culture medium is an important factor with significant impact on biosynthesis of valuable metabolites and its optimal level may be different for each plant species.

Shinde et al. [7] found that the application of 200 mg L^−1^ yeast extract allowed the optimal production of daizein and genistein in hairy root cultures of *Psoralea corylifolia* L. Other authors [8,9] noted that YE at 500 mg L^−1^ concentration was the most effective elicitor for production of metabolites such as hyoscyamine, scopolamine, isoflavones or noradrenaline in transformed root cultures of *Hyoscyamus niger*, *Pueraria candollei* and *Portulaca oleracea*, respectively. Slightly different observations were described by Hasanloo et al. [10] and Wawrosch et al. [11]. In their studies the stimulating influence of yeast extract on secondary metabolites was also confirmed, however higher levels of YE (2 g L^−1^ and 2.5 g L^−1^ respectively) had to be applied, whereas Bauer et al. [12] revealed that YE induced rosmarinic acid accumulation in two and diminished it in five out of 11 tested hairy root cultures of *Coleus blumei*.

Another factor effecting on the cellular response and deciding on the action of the yeast extract may be time exposure. The studies of Udomsuk et al. [8] and Hasanloo et al. [10] showed that 3 days of elicitation elevated the production of examined metabolites. Shorter time of incubation with YE (48 h) was suitable to 5-fold increase of noradrenaline level in hairy root cultures of *Portulaca oleracea* [9]. For the culture of transformed roots of *Salvia miltiorrhiza*, longer time of elicitation (7 days) was required to enhance the efficiency of rosmarinic and litospermic acid. Additionally, in this case the production of cryptotanshinone increased from 0.001% to 0.096% of dry weight [4].

Ginsenosides are triterpene saponins that are the main pharmacologically active ingredients of ginseng which is a perennial herbaceous plant belonging to the family *Araliaceae*. The major groups of ginsenosides are the Rb group and the Rg group derived from the 20(*S*)-protopanaxadiol and 20(*S*)-protopanaxatriol structures, respectively. Ginsenosides Rb1, Rb2, Rc, and Rd belong to the Rb group. The Rg group includes saponins Re and Rg1. A lot of in vivo and in vitro tests have indicated that the ginsenosides act on the immune, cardiovascular and nervous systems and that they have antistress, anticancer and neuroprotective activities [13,14,15,16,17]. The cultivation of ginseng requires a minimum 4 years before roots can be harvested. Moreover, field cultivation should be carried out under special conditions, where direct sunlight is blocked. Furthermore, ginseng is sensitive to different diseases and the agricultural treatments are needed to prevent them are expensive. For these reasons, original strategies using biotechnology techniques are being developed. They include using cell, adventitious and hairy root cultures as alternatives to field cultivation to produce the ginsenosides. Up till now the accumulation of ginsenosides in culture systems has been low. Therefore elicitation methods are used to optimize and improve ginsenoside production. For example, cell suspension cultures of *P. ginseng* were variously treated with jasmonic acid, its methyl ester derivative, salicylic acid, H_2_O_2_, vanadate, sorbitol oligogalacturonide acid, fungal cell wall and yeast extract [18,19,20,21,22,23]. A slightly different set of elicitors such as jasmonic acid, methyl jasmonate, ethephon, CuSO_4_, linolenic acid, oligosacharides, chitosan, Tween 80, fluorescent light and temperature were used in the elicitation process of *P. ginseng* adventitious and hairy root cultures [20,21,22,24,25,26,27,28,29,30,31,32,33]. As described above, most of the studies on the improvement of ginseng saponin production using elicitation methods were focused on *P. ginseng* cultures. Significantly fewer reports can be found about the effects of elicitation process with respect to in vitro cultures of other species of ginseng. Suspension culture of *P. notoginseng* was elicited using jasmonates, copper sulphate and calcium ions [34,35,36] while the cell cultures of *P. quinquefolium* were successfully treated lactoalbumin hydrolysate and methyl jasmonate. The analysis of current literature indicated that there are no studies on elicitation of transformed root culture of *P. quinquefolium*. Therefore, the aim of our research was to examine the effect of yeast extract and exposure time on ginsenoside biosynthesis by *P. quinquefolium* hairy roots cultivated in the shake flasks and in a nutrient sprinkle bioreactor. The choice of bioreactor is crucial to cultivate roots and hairy roots for metabolite production. Conventional bioreactors, which are generally used for cell suspension cultures cannot be used for hairy roots as they form root clumps which resist the percolation of oxygen. In this regard, a modified stirred tank bioreactor in which nutrient are sprayed on the roots is recommended [37,38].

## 2. Results

The present study investigated the effect of yeast extract on the saponin content in hairy root of *Panax quinquefolium* cultured in shake flasks and nutrient sprinkle bioreactor after 3 and 7 days of elicitation. The quantitative analysis was carried out for six ginsenosides: Rb1, Rb2, Rc, Rd (protopnaxadiol derivatives), and the Rg1, Re (protopanaxatriol derivatives).

### 2.1. Effect of the Concentration of Yeast Extract on Ginsenoside Accumulation in Shake Flask Cultures

**Figure 1:**
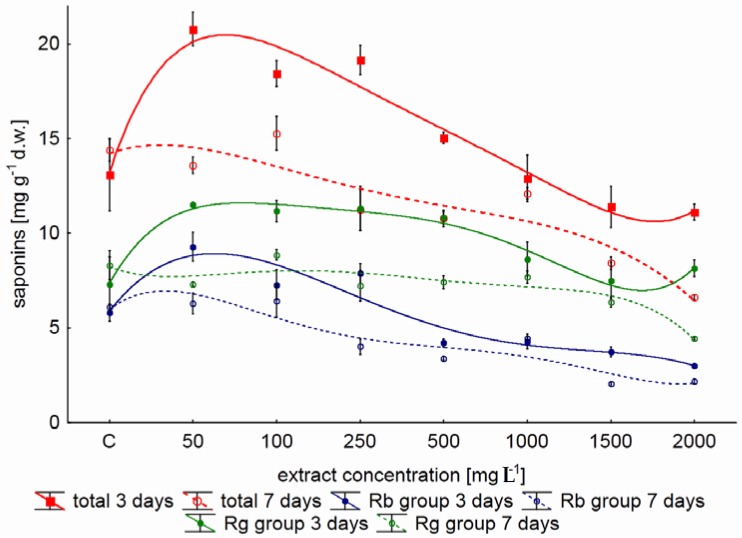
The influence of yeast extract concentration on ginsenoside level in hairy root cultures of *Panax quinquefolium*, cultivated in shake flasks after 3 and 7 days of elicitation.

In shake flask cultures, the highest total content of all tested saponins was determined in hairy root cultures after 3 days of elicitation with 50 mg L^−1^ yeast extract. The ginsenoside level reached 20.78 mg g^−1^ d.w. (Figure 1) and was increased by 37% compared to the control sample. Medium variants with addition of 100 and 250 mg L^−1^ yeast extract also resulted in an increase in the content of ginsenosides by 29% and 32%, respectively, with respect to untreated samples. Higher concentrations of the elicitor did not affect significantly or decreased the level of saponin. Moreover, experimental data showed that the prolonged exposure time (7 days) unfavorably affects the accumulation of ginseng saponins (Figure 1).

The analysis of changes in the levels of protopanaxatriol (Rg1 + Re) and protopanaxadiol (Rb1 + Rb2 + Rc + Rd) derivatives after a 3-day elicitation indicated that the content of Rg group saponins exceeded the saponin content of the group Rb. Furthermore, their level was higher in comparison to the control sample for all used concentrations of the elicitor, wherein 50 g L^−1^ YE was sufficient to achieve the maximum content of Rg1 + Re. In addition, the changes of the elicitor dose from 50 to 500 mg L^−1^ did not cause statistically significant changes in the level of Rg group saponins. YE added to the culture medium at higher amount (1000 mg L^−1^, 2000 mg L^−1^) decreased the accumulation of protopanaxartiol derivatives, but their level was still higher than in untreated attemps (Figure 1).

Another results were obtained for protopanaxadiol derivatives. The trend of changes in the content of saponins Rb group was similar to the changes observed for the total of six examined compounds. The level of protopanaxadiol derivatives was the highest in the medium containing 50 mg L^−1^ yeast extract. The obtained results showed that an inverse correlation was observed for the remaining concentrations of YE: when the amount of yeast extract in the medium increased, protopanaxadiol derivative content gradually reduced by half of their quantities (2.98 mg g^−1^ d.w. for 2000 mg L^−1^ YE) compared to untreated attempts (5.80 mg g^−1^ d.w.). Additionally, the results shown in Figure 1 also indicated that shorter time of elicitation was favorable for the accumulation of protopanaxadiol and protopanaxatriol derivatives.

**Figure 2:**
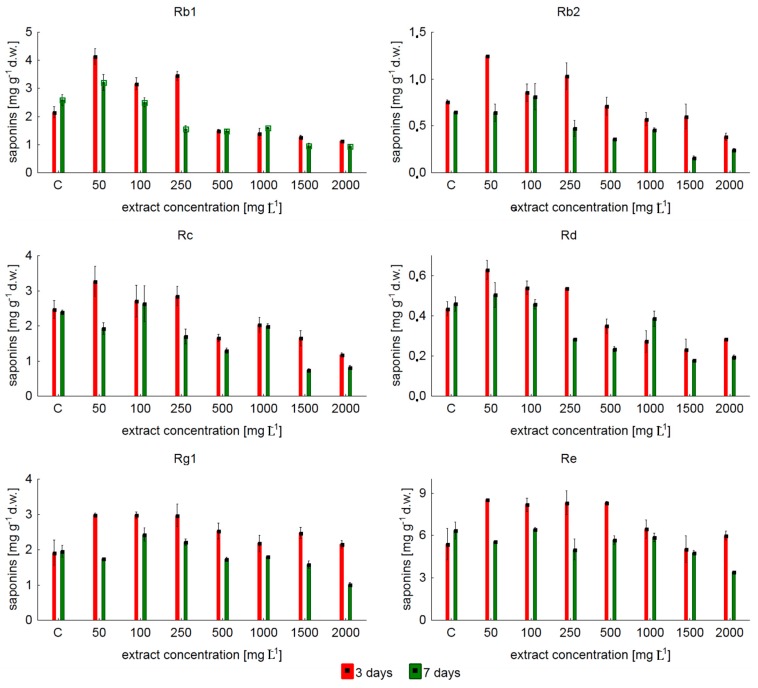
The influence of yeast extract concentration on individual ginsenoside levels in hairy root cultures of *Panax quinquefolium*, cultivated in shake flasks after 3 and 7 days elicitation.

The content of individual ginsenosides in hairy root cultures growing in shake flaks in relation to used yeast extract concentration in medium is presented in Figure 2.

The obtained results confirmed that the concentration of 50 mg L^−1^ yeast extract and 3-day time of exposure are the most appropriate conditions for increasing the Rb1, Rb2, Rc and Rd content. The level of these metabolites rose up in relation to the control sample by 92.7%, 64.77%, 32.76% and 44.57% respectively. The use of elicitor concentration greater than 250 mg L^−1^ resulted in reduction of the quantities of Rb1, Rb2, Rc and Rd ginsenosides under their level in untreated attempts.

Differently, as in the case of Rb1, Rb2, Rc and Rd saponins, for which one of the concentrations of yeast extract (50 mg L^−1^) clearly favored their accumulation, for Rg1 and Re more than one of the applied concentrations favours synthesis. The concentrations of YE suitable for stable synthesis of metabolite Rg1 were varied in the range of 50–250 mg L^−1^ and its content remained at the same level (2.97–2.98 mg g^−1^ d.w.) exceeding 1.5 times its amount in the control samples. The concentrations of YE suitable for stable synthesis of metabolite Re were 50–500 mg L^−1^. The obtained amount of Re ginsenoside varied slightly (8.19–8.53 mg g^−1^ d.w.) and accounted for 158% of its level indicated in the control trials. The elicitation process lasted three days was more favorable than 7-day exposure to yeast extract for the synthesis of all examined individual ginsenosides.

### 2.2. Effect of the Concentration of Yeast Extract on Ginsenoside Accumulation in Bioreactor Culture

**Figure 3:**
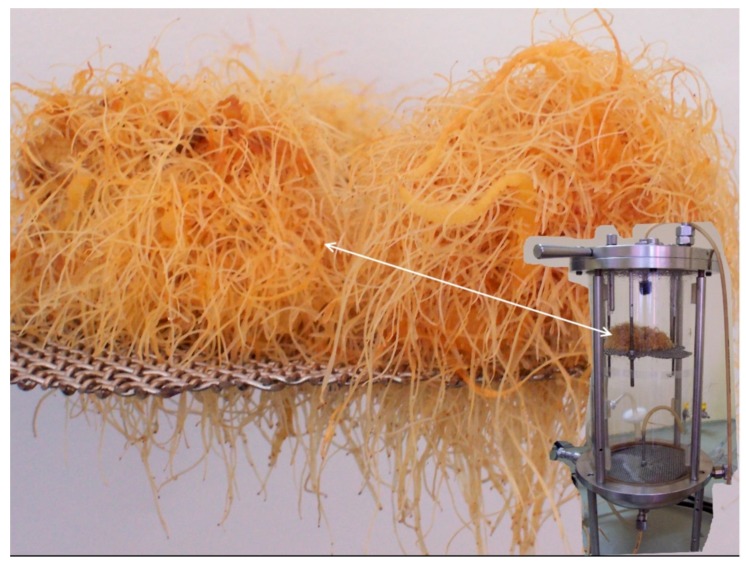
Bioreactor cultures of *P. quinquefolium* hairy roots.

To study the ginsenoside production in a bioreactor culture of *P. quinquefolium* hairy roots (Figure 3), yeast extract in a concentration of 50 mg L^−1^ was selected.

As demonstrated in the experimental data, 3-day elicitation stimulated the biosynthesis of examined ginsenosides. Longer time (7 days) of exposure caused the reduction of ginseng saponin accumulation. Total content of examined saponins achieved 32.25 mg g^−1^ d.w. and increased 2.46 fold in comparison to the control sample after 3-day elicitation (Table 1). The levels of ginsenosides belonging to Rb group and Rg group were 3.48 and 1.47 times higher than in untreated roots respectively. Saponin Rb1 quantitatively dominated and it accounted for 45.8% among protopanaxadiol derivatives and 31.91% of all identified metabolites (Table 1).

Similar results were found in relation to protopanaxatriol derivatives. According to our findings, the synthesis of these derivatives increased 1.47 times in comparison to the control sample. The individual levels of Rg1 and Re metabolites achieved 2.899 mg g^−1^ d.w. (54% increase) and 6.89 mg g^−1^ d.w. (43% increase) respectively.

The ratio of the derivatives (Rb group/Rg group) was used as an indicator for qualitative evaluation of ginseng material. In methanolic extracts obtained from in vitro culture, the ratios of protopanaxadiol derivatives to Rg ginsenosides were 0.81–0.86 and 1.86–2.25 respectively for shake flask and bioreactor cultures after elicitation of 50 mg L^−1^ yeast extract.

**Figure 4:**
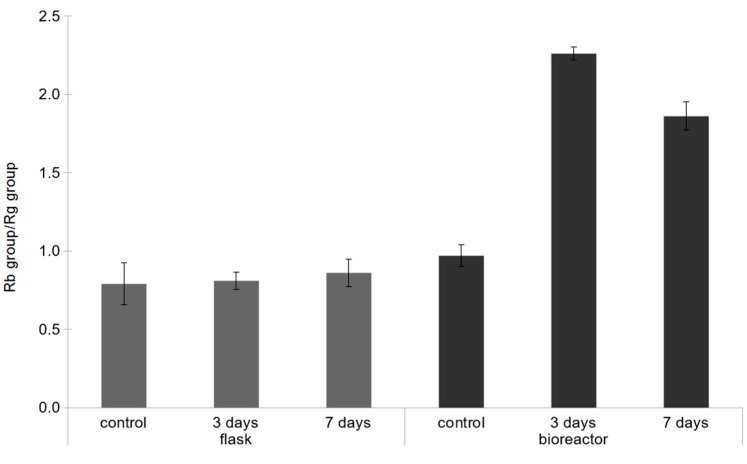
Rb group/Rg group parameter in the tested cultures after elicitation of 50 mg g^−1^ YE.

The obtained results indicated a significant increase in protopanaxadiol derivatives in bioreactor culture after treatment with elicitor. In control attempts Rb group/Rg group parameters were below 1, independently from the type of culture (Figure 4).

**Figure 5:**
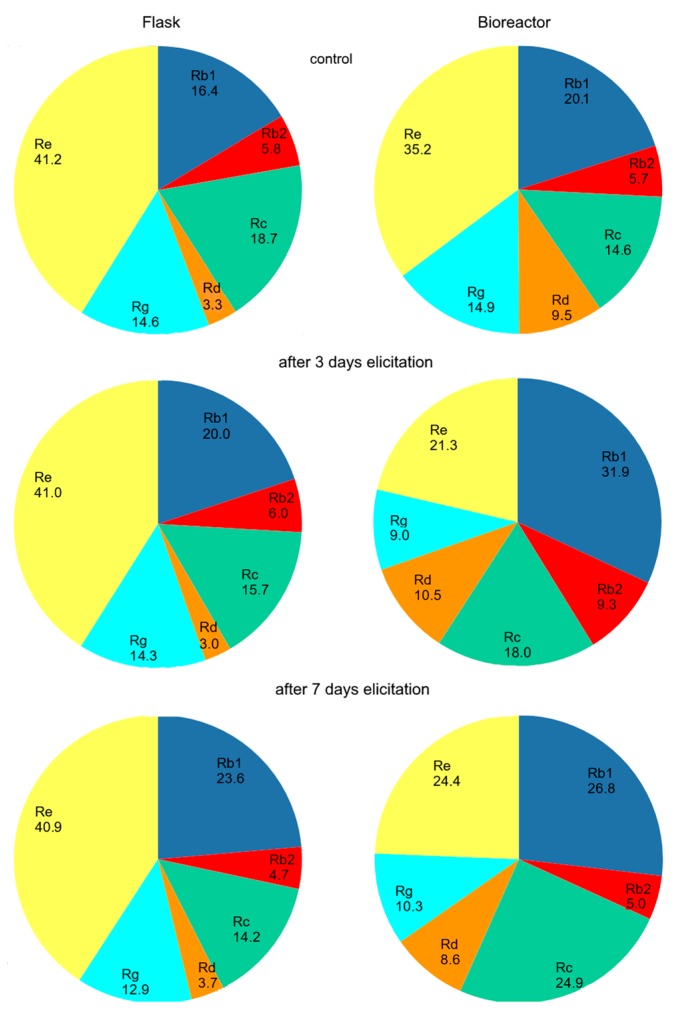
The percentage of individual ginsenosides in relation to all studied metabolites in hairy roots growing in the shake flasks and the nutrient sprinkle bioreactor.

The percentage of individual ginsenosides in relation to all studied metabolites was also established in hairy roots growing in the shake flasks and the nutrient sprinkle bioreactor. As Figure 5 demonstrates, the profiles of ginsenosides in untreated root cultures from the shake flasks and the bioreactor were different. The higher percentage of Rb1 and Rd and lower Re percentage were noticed in root cultures growing in the scale up. Comparing the profiles of individual saponins in bioreactor and shake flask cultures after a short elicitation, the increase of proportion of all protopanaxadiol derivatives and the reduction of the share of protopanaxartiol derivatives were found.

## 3. Discussion

Analyzing the current literature, we have not found any publication concerning the effect of yeast extract concentration on the production of ginsenosides in hairy roots of *P. quinquefolium*. Therefore, the purpose of this study was to determine the influence of different concentrations of this elicitor and varied exposure time on the saponin content in transformed roots of American ginseng cultured in the shake flasks and the nutrient sprinkle bioreactor.

Our findings indicate that the best variant of yeast extract concentration for efficient biosynthesis of ginsenoside in hairy root cultures of *P. quinquefolium* is 50 mg L^−1^ and 3 days of elicitation. Very few reports on the influence of YE on the saponin accumulation in in vitro cultures of ginseng can be found and those available concern only to research on *P. ginseng*.

Marsík et al. [39] applied for elicitation a 1000 mg L^−1^ concentration of yeast extract in adventitious root cultures of *P. ginseng* and observed that YE did not lead to an increase of ginsenoside content. These observations were similar to our results related to a similar concentration of YE. It is worth mentioning that the level of saponin content in Marsik et al. [39] investigation was 10 times lower compared to our findings received for 1000 mg L^−1^ of yeast extract regardless of the duration of elicitation process. The significant differences in saponin contents in adventitious root cultures of *P. ginseng* [39] and in hairy root cultures of *P. quinquefolium* in our study, were likely to be due to such factors as plant species, type of culture or length of elicitation.

In previous studies on suspension cultures of *P. ginseng* it was found that the ginsenoside content increased with increasing yeast extract concentration. Their maximum level was achieved at the highest concentration of yeast extract (3 g L^−1^), which was added to subculture on the day of inoculation [18]. These results are in contrast to our results for transformed roots of *P. quinquefolium*. The yield of saponin biosynthesis in hairy roots of *P. quinquefolium* was the highest at significantly lower concentrations of yeast extract (50 mg L^−1^) and the increase of YE to 2000 mg L^−1^ in the medium inhibited total ginsenoside production in the shake flask cultures.

The time of elicitor administration is very important for the efficient synthesis of ginsenosides. Lu et al. [18] reported that time of yeast extract treatment other than the day of inoculation did not cause the increase of saponin content in *P. ginseng* suspension cell cultures. According to the results of previous studies on the dynamic of saponin biosynthesis in these cultures [40] in hairy roots of *P. quinquefolium* the elicitor was added during stationary phase.

Yeast extract was used to improve secondary metabolite production in the culture of transformed roots of many plant species [4,8,9,10]. The maximum level of genistein and daidzein in the hairy roots of *Psoralea corylifolia* was achieved using 200 mg L^−1^ yeast extract concentration [7]. Meanwhile, significant increase in the levels of isoflavones, scopolamine and hyoscyamine in relation to the control was attained at a concentration of 500 mg L^−1^ YE in transformed roots of *Pueraria candollei* and *Hyoscyamus niger* [8,41]. There are also studies that state that higher yeast concentration like 2 or 5 g L^−1^ are suitable to effective yield enhance of leolignin and rosmarinic acid in cultures of *Edelweiss* and *Coleus blumei* [11,12]. It is in contrast to our findings, where the optimal amount of yeast extract for the efficient synthesis of ginseng saponin was only 50 mg L^−1^. On the other hand, Wilczańska-Barska et al. [42] and Srivastava and Srivastava [43] also successfully elicitated hairy root cultures applying low concentrations of yeast extract. The results by Wilczańska-Barska et al. [42] were in line with those obtained in this study and indicated that among several concentrations of yeast extract, the level of 50 mg L^−1^ is the most efficient at an acteoside and flavone production in *Scutellaria lateriflora* transformed root cultures.

Moreover, most of reports previously quoted confirmed our observations obtained for *P. quinquefolium* root cultures that increasing the elicitor concentration above the optimum value inhibited the production of examined compounds.

Another factor which can meaningfully influence the obtainable contents of metabolites, is the time of incubation with the elicitor. Our findings show that the 3-day elicitation better affects the ginsenosides production in transformed roots of American ginseng than a 7-day exposure time. The similar observation were demonstrated by Udomsuk et al. [8] who investigated *Pueraria candollei* hairy root cultures. The cultures of *Brugmansia candida* [3] responded the increment of intracellular content of scopolamine and hyoscyamine after 2 days of exposure to yeast extract. In the contrast to above investigations, the results of experiments conducted by Wilczyńska-Barska et al. [42] revealed that the production of acteoside was comparable independently of elicitor exposure time (7 and 14 days of treatment with yeast extract).

**Scheme 1:**
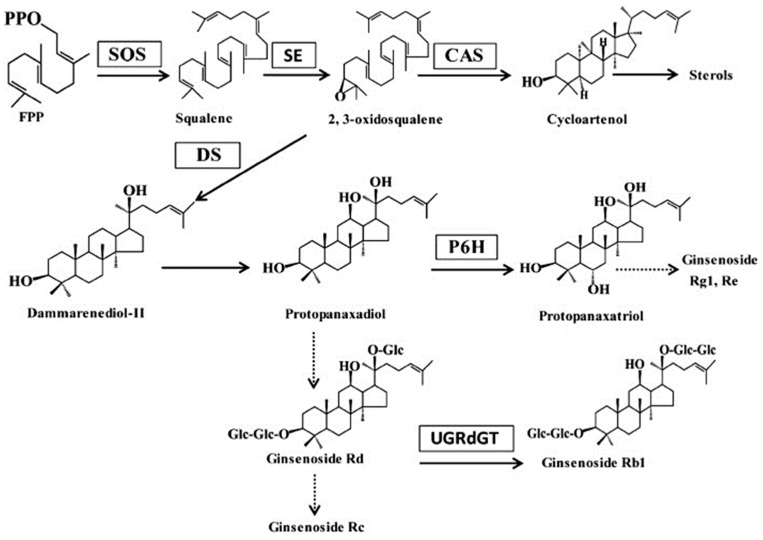
Proposed pathways for ginsenoside biosynthesis in *P. ginseng*. FPP-farnesyl diphosphate, SQS—squalene synthase, SE—squalene epoxidase, DS—dammarenediol-II-synthase, Glc—3-d-glucopyranosyl, P6H—protopaxadiol 6-hydroxylase; UGRdGT—UDGP-ginsenoside Rd glucosyltransferase [45].

To our knowledge, this is the first study referring to the improvement of biosynthesis of ginsenosides of *P. quinquefolium* in the bioreactor under the influence of elicitation by yeast extract. The results indicate differences in the profile of saponins in the flask and bioreactor root cultures. In bioreactor cultures the content of individual ginsenosides belonging to Rb group was higher in comparison to flask cultures. Meanwhile, protopanaxatriol derivatives as Re metabolites was accumulated in higher amount in flask cultures. The obtained findings may be due to the differences between bioreactor and shake flask processes. The differences mainly concern the nutrient supply and the changes in gas phase composition or the concentration of gaseous components (oxygen, carbon dioxide, ethylene and others). It is probable that these differences can influence the dynamics of ginsenoside biosynthesis and promote the pathway towards protopanaxadiol derivatives. Yue et al. [44] stated that protopanaxadiol 6-hydroxylase (P6H) is a key enzyme that determines the conversion of ginsenoside aglycone protopanaxadiol into protopanaxatriol. The activity of P6H was dependent on NADPH and molecular oxygen. Moreover, it was inhibited by carbon monoxide. Yue et al. [44] also demonstrated that the induction of protopanaxadiol 6-hydroxylase entailed the increase of protopanaxatriol-type ginsenoside content. In the light of this research [44], it can be supposed that activity of P6H has weaker activity in hairy root cultures of *P. quinquefolium* cultivated in bioreactor than in shake flask. In this case the lower level of protopanaxatriol, that can be converted to ginsenosides of Rg group, would cause the lower content of metabolite Re in bioreactor cultures. In the other hand, a very important role in the ending step of ginsenoside biosynthesis also plays the glucosyltransferases. These enzymes are necessary to synthesize specific ginsenosides. The results present in this paper can suggest that the activity of the glucosyltransferases, which lead to the synthesis of protopanaxadiol derivatives, was probably higher in bioreactor cultures. However, this conclusion is only a hypothesis because up till now these glucosyltransferases as well as the factors that regulate their activity have not been fully identified and characterized yet (Scheme 1).

Some studies have reported a significant reduction in metabolite production after transferring hairy root cultures from flasks to a bioreactor [46,47]. In this investigation the content of ginsenosides was higher in bioreactor than in flask cultures with added 50 g L^−1^ YE after 3 day elicitation. Similarly to our investigation, Eibl et Eibl [48] also showed that the ginseng saponin production was significantly higher in 2 L wave system than in shake flasks of hairy root cultures of *P. ginseng*.

After a review of previous research on the impact of YE on the production of secondary metabolites, it can be confirmed that yeast extract can stimulate their biosynthesis. The researchers suppose that the effect of yeast elicitor may be associated with the presence of some cations in it such as Zn, Ca, Co, which act as elicitors of abiotic stresses [43]. In the current literature it is difficult to find a hypothesis that accurately explains the mechanism of action of yeast extract on the synthesis of ginseng saponins. It is possible that the effects which have been observed in our research result from the metal ions contained in the yeast extract, which can probably act as abiotic elicitors, as suggested by Srivastava and Srivastava et al. [43]. On the other hand, the presence of amino acids, vitamins and minerals in yeast extract complex may play a role in the regulation of enzyme activity in the final stages of the biosynthesis of ginsenosides. So far there is no empirical or bioinformatic data that would characterize the enzymes involved in ginsenoside biosynthesis with respect to their activation or inhibition under influence of vitamins or aminoacids. Hence, the possible impact components of yeast extract on enzymes activity can be considered only hypothetically.

It seems that the activity of yeast extract on the secondary metabolite accumulation as a result of the presence of amino acids in it, can be also explained on the genetic level. As Kimball and Jefferson reported [49], amino acids can regulate multiple processes related to the gene expression including modulation of the function of the proteins that mediate messenger RNA (mRNA) translation. On the other hand, RNA blot analysis showed that farnesyl diphosphate synthase (FPS) and isopentenyl pyrophosphate isomerase (IPPI) gene coding enzymes involved in ginseng saponin biosynthesis were up-regulated by yeast extract, as a complex [50].

Elicitation strategies were used by many authors to enhance the levels of ginseng saponins in different in vitro cultures of *Panax* spp. such as cell suspensions, adventitious and hairy roots [51]. The most common and widely used elicitor for improvement of ginsenoside biosynthesis is methyl jasmonate (MeJa). MeJa has been found to enhance the transcriptional activity of key enzymes as farnesyl diphoshate, squalene synthase, squalene epoxidase or dammarenediol II—synthase in the pathway of ginsenoside biosyntesis [30]. For example, Palazon et al. [26] and Wang et al. [32] stated that total saponin content increased in response to MeJa in hairy and adentitious root cultures of *P. ginseng* respectively. The highest total ginsenoside content was obtained by use of 22 [26] and 10 mg L^−1^ [32] of this elicitor and was 4 and 4.76-fold greater than in the control group. On the contrary to these results, 250 mg L^−1^ chitosan treatment of hairy roots cultures of *P. ginseng* had a negative effect. Significant reduce of ginsenosides content in all tested root lines was observed [26].

## 4. Materials and Methods

### 4.1. Hairy Root Culture

The experiments were carried out with the hairy root culture of *Panax quinquefolium*. The hairy root cultures were obtained by *Agrobacterium rhizogenes* ATCC 15834 transformation according to the previously described method [40].

### 4.2. Shake Flasks Cultures

Hairy root cultures grow in the 300 mL shake Erlenmeyer flasks with 80 mL of hormone-free B-5 medium [52] containing 30 g L^−1^ sucrose. They were performed in the 250-mL shake flask on rotary shaker (100 rpm) in darkness at 26 °C ± 2 °C. The average inoculum size was about 300 mg fresh weight (f.w.) and 28.9 mg dry weight (d.w.).

### 4.3. Hairy Roots in the Nutrient Sprinkle Bioreactor

Bioreactor cultures were performed in the 10-dm^3^ nutrient sprinkle bioreactor (Figure 3) containing 1.5 L Gamborg medium [52]. An AG CH-8604 Volketswill peristaltic pump (Chemap, Mannedorf, Switzerland) was used to provide recirculation, as well as doses of B-5 medium supplemented with 30 g L^−1^ sucrose, through a dispersal nozzle to the roots, which were placed on stainless steel, and then turned to the bottom reservoir. The operating time of the pump was 30 s, supplying approximately 45 mL of medium, and the breaks in medium supply were 60 s. The nutrient was kept at a constant temperature by a Thermomix MM thermostat (B. Braun Biotech International, Melsungen, Germany). The bioreactor was inoculated with roots from five shake flask cultures, of approx. 25 g fresh weight and 2.15 g dry weight. The incubation was carried out at a temperature of 26 °C ± 2 °C, in the dark, for 5 weeks.

### 4.4. Elicitation Process

The medium was supplemented with yeast extract (BTL, Łódź, Poland) at concentrations of 0, 50, 100, 250, 500, 1000, 1500, 2000 mg L^−1^ in shake flask culture. The extract was added at 28 day of culture, when the culture was in the stationary phase of growth. The concentration of YE, which the best stimulated ginsenoside production in shake flask cultures, was selected to the studies in nutrient sprinkle bioreactor. The extract was given at 28 day of bioreactor culture as in the experiment with shake flask cultures. After 3 and 7 days of elicitation, hairy roots cultured in the shake flasks and bioreactor were harvested and ginsenoside content was determined.

### 4.5. Ginsenoside Content Determination

#### 4.5.1. Sample Preperation

Fresh roots, after drying on absorbent paper, were dried at room temperature and were subjected to the ginsenoside extraction process and HPLC analysis. Samples of 1 ± 0.1 g of dry raw material were placed in the 250 mL flasks. They were extracted three times with 50 mL of 80% methanol for 30 min at solvent boiling temperature under a reflux condenser. The combined methanol extracts were evaporated to dryness in a vacuum evaporator under lowered pressure at 60 °C. The flasks with dried residues was placed in a desiccator filled with drying agent. The dried methanolic extract was weighed and purified using the SPE column.

#### 4.5.2. Solid Phase Extraction (SPE) Steps

In the first step, a SPE column with octadecyl (C18) as reverse phase packing was rinsed with 10 mL 100% methanol of HPLC grade (J.T. Baker, Deventer, The Netherlands) and 10 mL HPLC water (J.T. Baker) to prepare the column to obtain the sample. Methanolic extracts were dissolved in 5 mL 50% HPLC grade methanol. The samples (4.5 mL) were placed on the column. In the next step, the columns were rinsed with 10 mL HPLC water and 10 mL 30% HPLC methanol to selectively remove impurities. Ginsenosides were selectively eluted using 10 mL 100% methanol of HPLC grade and were evaporated until dryness in a vacuum evaporator under lowered pressure at 60 °C. The dried extracts were used for HPLC analysis.

### 4.6. Quantitative Analysis of Ginsenosides

#### 4.6.1. Standard Solution and Standard Curve Preparation

Ginsenosides Rb1, Rb2, Rc, Rd, Re, Rg1 were purchased from C. Roth GmbH+Co (Karlsruhe, Germany). A standard of the ginsenosides Rb1, Rb2, Rc, Rd, Re and Rg1 were accurately weighed and dissolved in methanol (HPLC grade) and analyzed by the HPLC methods. Different volumes of mixed standard stock was given on HPLC apparatus to create standard curves. The peak area of each ginsenoside standard was recorded. The standard curve was calculated according to the peak area and concentration. Limit of detection (LOD) and limit of quantification (LOQ) were also acquired by testing noise-signal rations, respectively. The detailed regression equation and validation data of each standard are listed in Table 2.

#### 4.6.2. HPLC Analysis of Ginsenosides

Dried extracts were dissolved in 1 mL of methanol (HPLC grade) and filtered through 0.2 μm pore diameter Millex^®^-FG Hydrophobic Fluoropore filter (PTFE) (Merck, Darmstadt, Germany) and were entered into the liquid chromatography system consisting of an Agilent Technology 1200 apparatus (Agilent Technology, Santa Clara, CA, USA), a ZORBAX Eclipse XDB-C18 (150 × 4.6 mm, 5 µm) column, Quat Pump and a UV-VIS DAD type detector, and autosampler set combined with a Agilent ChemStation 2001–2010 software system.

Two different mixtures of acetonitrile with water were used as the eluent. An acetonitrile to water ratio of 30:70 was used for determination of ginsenosides Rb1, Rb2, Rc, Rd (flow rate 2 mL min^−1^, analysis time 45 min.), and an 18:82 ratio was used for determination of ginsenosides Rg1 and Re (flow rate 3 mL min^−1^, analysis time 40 min.). Ginsenoside detection was performed at a wavelength of 203 nm. Ginsenosides were quantified (mg g^−1^ d.w.) by comparing retention time and peak areas between standards and samples.

#### 4.6.3. Statistical Analysis

All treatments were performed in triplicate. Data was analysed using the Kruskal-Wallis test. Any relationships were considered significant at *p* ≤ 0.05. Statistica Version 12.5 software was used for all statistical analyses (STATSoft, Tulsa, OK, USA).

## 5. Conclusions

This study evaluated the effect of different concentrations of yeast extract on the production of ginsenosides in transformed root cultures of *Panax quinquefolium* in the shake flasks and the nutrient sprinkle bioreactor depending on the exposure time of elicitor. The results showed that the 3-day elicitation promoted ginsenosides synthesis more than 7-day time of exposure both in root cultures grown in the shake flasks and the bioreactor.

The yeast extract at a concentration of 50 mg L^−1^ proved to be optimal for efficient biosynthesis of ginsenosides. The increase of the total saponin content was greater in the shake flask experiments and the bioreactor by 55% and 140%, respectively, compared to saponin content in the untreated samples. The concentration of 50 mg L^−1^ yeast extract also contributed to the synthesis of individual saponins from the Rb group. For ginsenosides of the Rg group, yeast extract the concentration range of 50–250 mg L^−1^ can be determined as the most suitable for Rg1 and Re production. Saponin Re quantitatively dominated in shake flasks cultures, whereas in bioreactor cultures it was metabolite Rb1. Additionally, the elicitation of yeast extract resulted in a significant increase in the synthesis of protopanaxadiol derivatives in the fermenter.

## Figures and Tables

**Table 1:** Ginsenoside content after elicitation of 50 mg g^−1^ YE in bioreactor cultures of *P. quinquefolium*.

Ginsenosides [mg g^−1^ d.w.] ± SE									
	Rb1	Rc	Rb2	Rd	Rg1	Re	Rb group	Rg Group	Total
C	2.66 ± 0.03 ^a^	1.96 ± 0.05 ^a^	0.83 ± 0.06 ^a^	1.01 ± 0.04 ^a^	1.88 ± 0.03 ^a^	4.79 ± 0.09 ^a^	6.46 ± 0.03 ^a^	6.67 ± 0.12 ^a^	13.13 ± 0.14 ^a^
3d	10.29 ± 0.27 ^b^	5.79 ± 0.07 ^b^	2.99 ± 0.07 ^b^	3.38 ± 0.13 ^b^	2.90 ± 0.14 ^b^	6.89 ± 0.11 ^b^	22.46 ± 0.82 ^b^	9.79 ± 0.25 ^b^	32.25 ± 0.44 ^b^
7d	3.02 ± 0.14 ^a^	2.80 ± 0.12^c^	0.58 ± 0.04^a^	0.97 ± 0.04 ^a^	1.16 ± 0.01 ^c^	2.74 ± 0.04 ^c^	7.37 ± 0.03 ^a^	3.91 ± 0.05 ^c^	11.28 ± 0.38 ^a^

C—control, 3d—3 day elicitation, 7d—7 day elicitation, SE—standard error. Values in each column marked with the same letter do not differ significantly according to the Kruskall-Wallis test (*p* ≤ 0.05).

**Table 2:** The standard curve of ginsenosides.

Ginsenosides	Regresion Equation	r^2^	LOD (µg mL^−1^)	LOQ (µg mL^−1^)
Rb1	*y* = 136.13*x* − 1.55	0.9991	2.01	6.63
Rb2	*y* = 124.39*x* − 3.57	0.9984	2.88	9.50
Rc	*y* = 117.58*x* − 1.99	0.9997	1.35	4.46
Rd	*y* = 166.19*x* − 0.37	0.9987	2.00	6.60
Rg1	*y* = 156.21*x* − 2.26	0.9998	0.60	1.98
Re	*y* = 143.98*x* − 0.64	0.9994	1.37	4.52

*x* represents the concentration of ginsenosides; *y* represents the peak area of ginsenoside, LOD—limit of detection, LOQ—the limit of quantification.
